# Prolonged treatment with N-acetylcystine delays liver recovery from acetaminophen hepatotoxicity

**DOI:** 10.1186/cc7782

**Published:** 2009-04-09

**Authors:** Runkuan Yang, Keita Miki, Xin He, Meaghan E Killeen, Mitchell P Fink

**Affiliations:** 1 Department of Critical Care Medicine, University of Pittsburgh Medical School, 3550 Terrace Street, Pittsburgh, PA 15261, USA; 2 Department of Pathology, University of Pittsburgh Medical School, 3550 Terrace Street, Pittsburgh, PA 15261, USA

## Abstract

**Introduction:**

Acetaminophen (APAP) toxicity is the most common cause of acute liver failure in the US and Europe. Massive hepatocyte necrosis is the predominant feature of APAP-induced acute liver injury (ALI). Liver regeneration is a vital process for survival after a toxic insult, it occurs at a relative late time point after the injurious phase. Currently, N-acetylcysteine (NAC), a glutathione precursor, is the antidote for acetaminophen overdose. However, NAC is effective only for patients who present within hours of an acute overdose, and is less effective for late-presenting patients. It is possible that in delayed patients, previously reduced endogenous glutathione (GSH) level has restored and prolonged treatment with NAC might be toxic and impair liver regeneration. Therefore, we hypothesize that prolonged treatment with NAC impairs liver regeneration in ALI induced by APAP.

**Methods:**

ALI was induced in C57BL/6 male mice by a single dose of APAP (350 mg/kg) by intraperitoneal injection. After two hours of APAP challenge, the mice were given 100 mg/kg NAC dissolved in 0.6 mL saline, or saline treatment every 12 hours for a total of 72 hours.

**Results:**

Seventy-two hours after APAP challenge, compared with saline treatment, NAC treatment significantly increased serum transaminases (alanine transaminase/aspartate aminotransferase), induced evident hepatocyte vacuolation in the periportal area and delayed liver regeneration seen in histopathology. This detrimental effect was associated with reduced hepatic nuclear factor (NF)-κB DNA binding and decreased expression of cell cycle protein cyclin D1, two important factors in liver regeneration.

**Conclusions:**

Prolonged treatment with NAC impairs liver regeneration in ALI induced by APAP.

## Introduction

Acetaminophen (APAP) toxicity is the most common cause of acute liver failure in the US and Europe [1]; however, the underlying mechanisms of APAP-induced hepatotoxicity are still not completely understood. The toxic response to APAP is triggered by a highly reactive metabolite, N-acetyl-p-benzoquinone imine (NAPQI), which reacts with and depletes glutathione (GSH), after which it forms covalent adducts and initiates mitochondrial oxidative stress [2,3]. This increases the membrane permeability transition and causes the collapse of the mitochondrial membrane potential, which results in a diminished mitochondrial capacity to synthesize ATP [4], and ATP depletion leads to cell death. Massive necrosis of the hepatocyte is a characteristic feature of APAP-induced acute lung injury (ALI) [5].

Liver regeneration is a vital process for survival after a toxic insult [6,7]. Regeneration ensures the replacement of necrotic cells and the full recovery of organ function. As hepatocytes are mostly in a quiescent state (G_0_), the regeneration process requires entry into the highly regulated cell cycle [8]. The induction of cyclin D1 is the most reliable marker for cell cycle (G_1 _phase) progression in hepatocytes. Once hepatocytes express cyclin D1, they have passed the G_1 _restriction point and are committed to DNA replication [8].

Many factors can influence liver regeneration. Currently, nuclear factor (NF)-κB is thought to play a major role in the initiation of liver regeneration after cell or tissue loss (such as by hepatotectomy) [8]. In addition, nutrients and metabolic status can also influence regeneration, because APAP induces massive hepatocyte necrosis. After the loss of a large number of parenchymal cells, the metabolic work of surviving hepatocytes is increased and more ATP is needed for maintaining homeostasis and regeneration [8].

Currently, N-acetylcysteine (NAC), a GSH precursor, is the antidote for APAP overdose [9]. However, this antidotal therapy is effective only for patients who present within hours of an acute overdose, and is less effective for late-presenting patients [9,10]. There are two possibilities: delayed treatment with NAC is not effective when massive hepatonecrosis has occurred; at a late hour, liver regeneration becomes evident and plays a crucial role in liver recovery; however, delayed NAC treatment does not maintain the proliferation of primary hepatocytes [11]. In addition, there is evidence showing that reduced endogenous GSH concentration gradually gets back to a normal level at the late time point [12], after which continuous treatment with NAC may no longer replenish endogenous GSH. Instead, it might interfere with glucose metabolism, because NAC is involved in glucose and mitochondrial tricarboxylic acid (TCA) metabolisms [13]; and a high dose of NAC (600 to 1200 mg/kg) impairs liver glucose metabolism [13]. The liver is responsible for the metabolism of carbohydrate, lipid and protein; these processes are all interlinked [14], therefore, prolonged treatment with a high dose of NAC may interfere with hepatic normal metabolic functions and impairs liver recovery from APAP hepatotoxicity.

At present, NAC is also used to treat non-acetaminophen-induced hepatotoxicity [15,16]. However, there are limited data available on the efficacy and safety of NAC. The doses vary from 100 mg/kg/20 hours to 300 mg/kg/24 hours in patients; the median duration of NAC administration in children with non-APAP-induced ALI is five days (range = 1 to 77 days) [16]. In an amatoxin (a toxic peptide from poisoning mushroom) induced hepatotoxicity model, NAC was administered at a dosage of 1200 mg/kg and repeated every four hours at a dosage of 600 mg/kg for a total of 48 hours. The NAC-treated mice have higher serum alanine transaminase (ALT)/aspartate aminotransferase (AST) than the amatoxin-challenged mice treated with saline [15], this indicates that prolonged treatment with a high dose of NAC might be toxic.

Based on this information, we hypothesized that NAC might impair liver regeneration in ALI induced by APAP. To evaluate this idea, ALI was induced in mice by APAP intraperitoneal injection, and the mice were observed over a 72-hour period. Compared with saline treatment, prolonged treatment with NAC increased serum ALT/AST and also induced evident hepatocyte vacuolation and delayed liver regeneration as shown by histopathology; This detrimental effect was associated with reduced hepatic NF-κB DNA binding and decreased expression of cell cycle protein cyclin D1, two important factors in liver regeneration. In conclusion, prolonged treatment with therapeutic dose of NAC impairs liver regeneration in ALI induced by APAP, reevaluation of optimal doses and duration of NAC therapy is needed.

## Materials and methods

### Materials

All chemicals were purchased from Sigma-Aldrich Chemical Co. (St. Louis, MO, USA) unless otherwise noted.

### Animal model and experimental groups

This research protocol complied with the regulations regarding the care and use of experimental animals published by the National Institutes of Health and was approved by the Institutional Animal Use and Care Committee of the University of Pittsburgh Medical School. Male C57BL/6 mice weighing 20 to 25 g (Jackson Laboratories, Bar Harbor, ME, USA) were used in this study. The animals were maintained at the University of Pittsburgh Animal Research Center with a 12-hour light-dark cycle and free access to standard laboratory food and water. The animals were fasted over night prior to the experiments.

#### Experiment A

ALI was induced by a single dose of APAP (350 mg/kg dissolved in 1 mL sterile saline) administered by intraperitoneal injection. APAP-challenged mice were then randomized into the NAC (n = 7) group or the saline group (n = 7). Six mice injected with saline not containing APAP served as a control group. Two hours after APAP administration, each group was given the following treatments every 12 hours for a total of 72 hours: 100 mg/kg NAC dissolved in 0.6 mL sterile saline for the NAC group, and 0.6 mL saline for the saline group and the control group. Seventy-two hours after APAP injection, all surviving mice in each group were anaesthetized with sodium pentobarbital (90 mg/kg intraperitoneally), and the following procedures were performed: blood was aspirated from the heart for the subsequent measurements of ALT and AST; the left lobe of the liver was harvested for pathology (H&E staining); and the right lobe of the liver was harvested and frozen for measurement of hepatic NF-κB DNA binding by electrophoretic mobility shift assays (EMSA) and hepatic tissue cyclin D1 expression by western blot.

#### Experiment B

Three separate groups of mice were treated as described above with the exception that the treatment period was only 24 hours (n = 6 for each group).

### Plasma aminotransferase measurements

Plasma levels of AST and ALT were measured at 37°C with a commercially available kit (Sigma Diagnostic, St Louis, MO, USA).

### Histological analysis

Consecutive sections (5 μm) of paraffin-embedded liver were prepared for H&E staining. The percentage of necrosis was estimated by evaluating the number of microscopic fields with necrosis compared with the entire cross-section. In general, necrosis was estimated at low power (×100) and questionable areas were evaluated at higher magnification (×200 or ×400). The pathologist (XH) evaluated all histological sections in a blinded fashion. Inflammatory cell infiltration results were scored semi-quantitatively by averaging the number of inflammatory cells per microscopic field at a magnification of 200×. Five fields were evaluated per tissue sample, and six animals in each group were examined.

### Tissue myeloperoxidase

Neutrophil infiltration was measured at 72 hours by determining myeloperoxidase (MPO) activity in liver tissue homogenates and was used as an index of neutrophil infiltration in all groups. At the time of sample determination, liver tissue samples (400 mg) were harvested and snap frozen immediately in liquid nitrogen and stored at -70°C until analysis was performed. Samples were homogenized in suspension buffer (50 mmol/L potassium phosphate/0.5% hexadecyltrimethylammonium bromide; pH 6.0), sonicated on ice, freeze-thawed twice, resonicated and then centrifuged for 15 minutes as 20,000 g at 4°C. Liver supernatants were heated for two hours at 60°C, and recentrifuged for 15 minutes at 20,000 g at 4°C.

To determine the MPO activity, 100 μL of the supernatants was incubated for three minutes at 37°C in a reaction solution containing 40% PBS, 8% N,N-dimethylformamide, 1.6 mmol/L 3,3', 5,5'-tetramethylbenzidine, 0.3 mmol/L hydrogen peroxide and 80 mmol/L sodium phosphate (pH 5.4). The reaction was stopped by adding ice-cold 800 mmol/L acetic acid solution (pH 3.0) and by placing the assay tubes on ice. Samples were read at 655 nm and total MPO activity calculated as the change in absorbency per minute per gram of tissue multiplied by the dilution factor. The levels were expressed as units per gram of tissue (U/g) [17].

### EMSA

NF-κB activation was determined by EMSA as previously described [18]. The gels were dried and exposed to Biomax film (Kodak, Rochester, NY, USA) at -70°C overnight with use of an intensifying screen.

### Western blot

Liver protein was extracted as previously described [19]. Equivalent amounts of protein were boiled in sample buffer and separated on 7.5% pre-cast SDS-PAGE (Bio-Rad, Hercules, CA, USA) and transferred to nylon membranes. Membranes were then probed with a specific antibody against cyclin D1 (Cell signaling Technology, Lexington, KY, USA) protein, visualized with an Enhanced Chemiluminescence substrate (ECL, Amersham Pharmacia Biotech, Piscataway, NJ, USA) and exposed to x-ray film according to the manufacturer's instructions.

### Statistical methods

Results are presented as means ± standard error of the mean (SEM). Continuous data were analyzed using student's t-test or analysis of variance followed by Fisher's least significant difference test. *P *values less than 0.05 were considered significant. Summary statistics are presented for densitometry results from studies using western blot for cyclin D1 expression but these results were not subjected to statistical analysis because the method employed was only semi-quantitative (n = 6).

## Results

### Prolonged treatment with NAC increases serum ALT/AST at 72-hour time point

**Figure 1:**
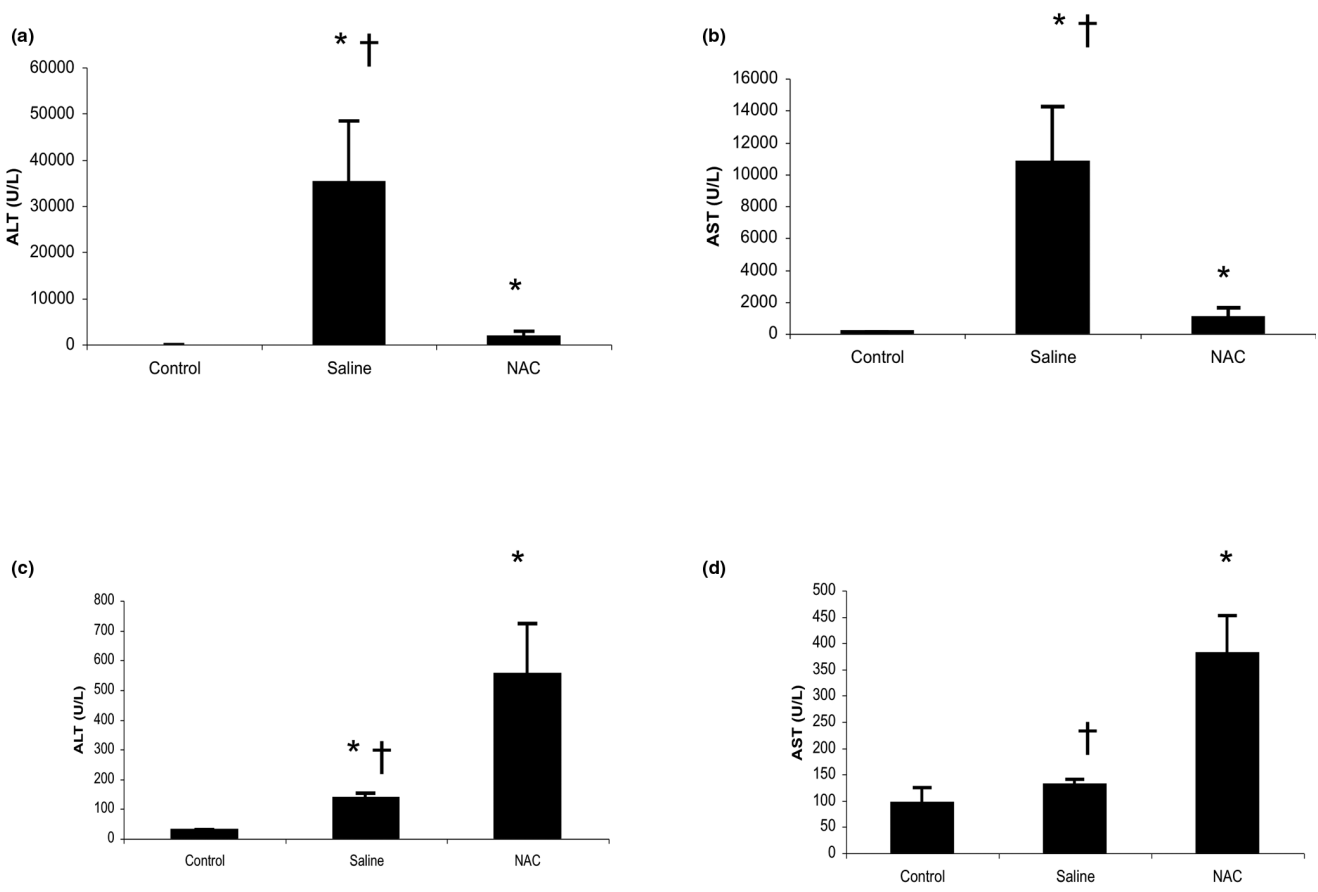
Effect of treatment with NAC or saline on serum ALT/AST in an APAP-induced ALI model. **(a, b) **Acute liver injury (ALI) was induced in C57Bl/6 male mice with a single dose of acetaminophen (APAP) (350 mg/kg) by intraperitoneal injection. Two hours after APAP injection, the animals were treated with 100 mg/kg N-acetyl-cysteine (NAC) dissolved in 0.6 mL saline or 0.6 mL saline every 12 hours. Alanine aminotransferase (ALT) and aspartate aminotransferase (AST) were measured 24 hours after APAP injection (n = 6 surviving mice for each group). Results are means ± standard error of the mean (SEM). * *P *< 0.05 versus control; † *P *< 0.05 vs. the NAC group. **(c, d) **Three separate groups of mice were used. ALI was induced as described above. Two hours after APAP challenge, the animals were given the same treatment every 12 hours for a total of 72 hours. ALT and AST were measured 72 hours after APAP injection (n = 6 to 7 surviving mice for each group). Results are means ± SEM. * *P *< 0.05 vs. control; † *P *< 0.05 vs. the NAC group.

Twenty-four hours after APAP injection, compared with saline treatment, NAC therapy significantly decreased serum concentrations of ALT/AST (Figure 1a, b). However, 72 hours after APAP challenge, compared with saline treatment, NAC therapy significantly increased serum ALT/AST concentrations (Figure 1c, d).

### Prolonged treatment with NAC impairs liver regeneration in histopathology

**Figure 2:**
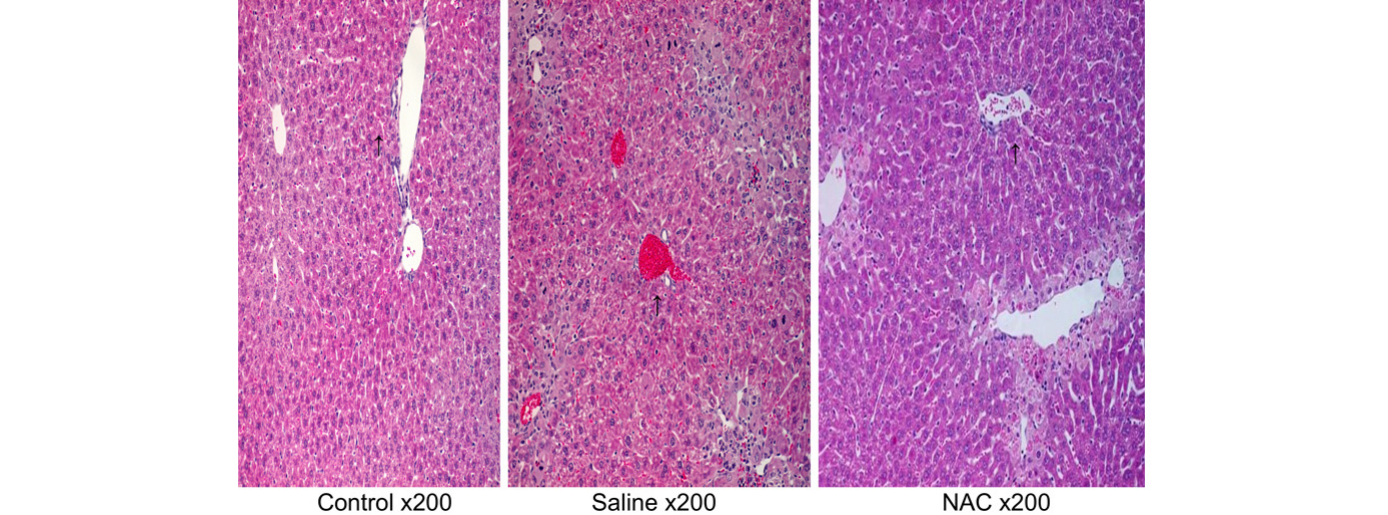
Effect of treatment with NAC or saline on pathology in mice with ALI. H&E staining was assessed 72 hour after induction of acute liver injury (ALI) or sham procedure (n = 6 for each group). Typical picture is shown. Arrows indicate periportal areas shown in Figure 3. NAC = N-acetyl-cysteine.

**Figure 3:**
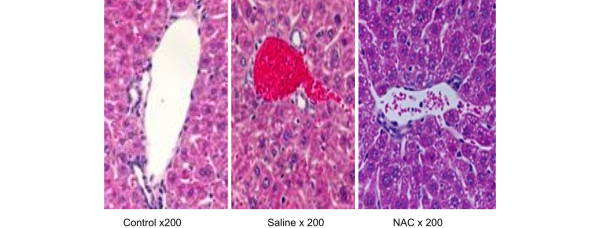
Prolonged treatment with NAC induces periportal hepatocytes vacuolation in mice with ALI. H&E staining was assessed 72 hour after induction of acute liver injury (ALI) or sham procedure (n = 6 for each group). Typical picture is shown. NAC = N-acetyl-cysteine.

In histological evaluation 72 hours after ALI induction, compared with control animals, saline-treated mice demonstrated a small number of scattered necrotic hepatocytes, evident regeneration and extensive infiltration of inflammatory cells (240 ± 40 per high power field, n = 6) in the centrilobular region. In contrast, NAC-treated mice demonstrated 10% necrotic hepatocytes and extensive inflammatory cell infiltration (250 ± 35 per high power field, n = 6) in the centrilobular region; however, no evident regeneration was seen (Figure 2; arrows indicate periportal areas shown in Figure 3). In addition, prolonged treatment with NAC also induced evident hepatocyte vacuolation in the periportal area (Figure 3).

### Prolonged treatment with NAC increases hepatic tissue MPO level

**Figure 4:**
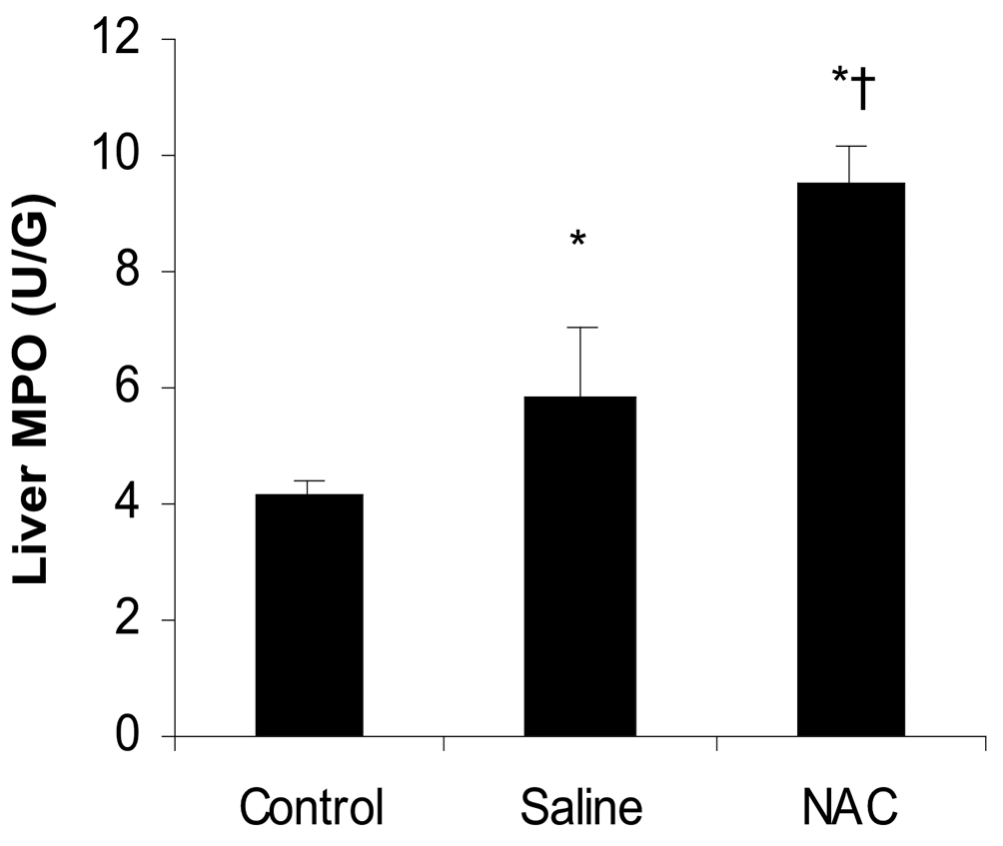
Effect of treatment with NAC or saline on hepatic MPO activity in mice with ALI. Liver myeloperoxidase (MPO) was assessed 72 hours after induction of acute liver injury (ALI) or sham procedure. Results are means ± standard error of the mean (SEM). * *P *< 0.05 versus control; † *P *< 0.05 vs. saline. NAC = N-acetyl-cysteine.

Tissue MPO activity was determined as an index of neutrophil infiltration after the APAP injection in the liver. Liver MPO activity values for the control group were 4.2 ± 0.29 U/g (Figure 4). Seventy-two hours after ALI, these values increased to 5.88 ± 1.19 U/g in the saline group. The liver MPO activity was significantly increased in the NAC therapy group, to a value of 9.5 ± 0.70 U/g (*P *< 0.05), when compared with the saline group (n = six for each group, data were shown as mean ± SEM).

### Prolonged NAC therapy decreases hepatic NF-κB DNA binding

**Figure 5:**
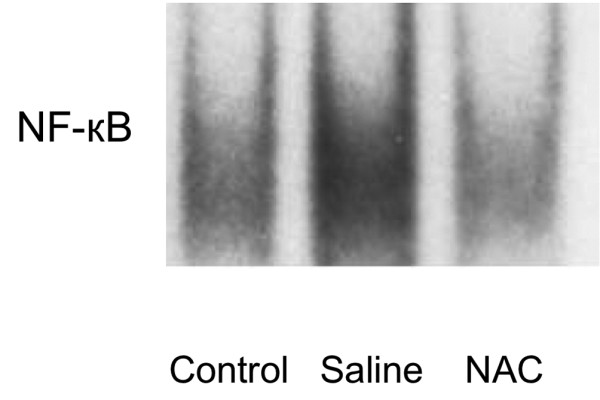
Effect of treatment with NAC or saline on NF-κB DNA binding in nuclear extracts prepared from hepatic tissue samples from mice with ALI. Nuclear factor (NF) κB DNA binding was assessed 72 hours after induction of acute liver injury (ALI) or sham procedure. The figure depicts results from five representative assays. Typical gels are depicted. NAC = N-acetyl-cysteine.

NF-κB is a pleiotropic transcription factor whose activation has been linked to inflammatory and destructive processes, as well as initiation of regenerative programs in the injured liver. Blockade of HMGB1 protects against ischemia-reperfusion (I/R)-induced liver injury; this protection is associated with increased NF-κB DNA binding activity [20]. Enhanced NF-κB activation is seen in mice that are protected from hepatic I/R following blockade of the HMGB1 receptor for advanced glycation end products [21]. Therefore, we examined the impact of APAP on activation of NF-κB 72 hours after APAP injection and tested the effect of NAC treatment. There was a low basal level of NF-κB DNA binding in the hepatic tissue samples in the control group. In the saline group, there was a marked increase in NF-κB DNA binding. Prolonged treatment of mice after APAP challenge with NAC clearly decreased NF-κB DNA binding relative to the degree observed in mice treated with saline (Figure 5).

### Prolonged treatment with NAC decreases hepatic cyclin D1 expression

**Figure 6:**
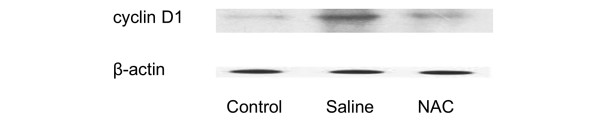
Effect of treatment with NAC or saline on the expression of cyclin D1 in the hepatic tissue. Western blot was performed using hepatic extracts prepared from tissues obtained 72 hours after acetaminophen injection. The figure depicts results from five representative assays. Typical gels are depicted. NAC = N-acetyl-cysteine.

The timely onset of tissue repair processes can limit liver injury and promote regeneration of lost tissue mass [7]. The induction of cyclin D1 is the most reliable marker for cell cycle (G_1_ phase) progression in hepatocytes [8]. Western blot was performed using whole-cell extracts prepared from liver tissue to assess expression of cyclin D1 in mice subjected to ALI or the control procedure. Cyclin D1 expression in the control group and the NAC group was minimal (Figure 6). In contrast, cyclin D1 expression was clearly observed in saline-treated animals at 72 hours after APAP administration, which indicates that prolonged treatment with NAC inhibits hepatic cyclin D1 expression.

## Discussion

The purpose of this study was to test the hypothesis that prolonged treatment with NAC impairs liver regeneration in ALI induced by APAP. The major and the novel findings of this investigation are: prolonged treatment with NAC increases serum ALT/AST and delays liver regeneration 72 hours after APAP administration; prolonged treatment with a therapeutic dose of NAC induces evident hepatocyte vacuolation around periportal area; the detrimental effect is associated with reduced NF-κB DNA binding; and prolonged NAC therapy significantly decreases the expression of cell cycle protein cyclin D1 in liver tissue.

In this study, prolonged NAC treatment demonstrated a large amount of hepatonecrosis in the centrilobular region and evident hepatocyte vacuolation in the periportal area; and also induced increased neutrophil infiltration as determined by measuring myeloperoxidase activity in total liver extracts. In addition, no evident regeneration was seen in the NAC group by histopathology. In contrast, saline treatment showed only a small number of scattered necrotic hepatocytes and evident regeneration. These data indicate that liver regeneration is delayed by prolonged NAC therapy and support the notion that timely onset of tissue repair processes can limit liver injury [7].

To elucidate the molecular basis of liver recovery in prolonged NAC therapy, we investigated its effect on the NF-κB signaling pathway because activation of NF-κB is linked strongly not only to the inflammatory response [20], but also to liver regeneration [8]. In addition, NF-κB is currently thought to play a major role in the initiation of liver regeneration after cell or tissue loss (such as partial hepatectomy) [8,22]. NF-κB activation also induces increased expression of survival genes, including BCL_XL _and A1 [23]. Our data suggested that prolonged treatment with NAC is associated with a detrimental effect characterized by reduced NF-κB DNA binding. Although NF-κB activation modulates inflammation [24], it is also known to protect hepatocytes from cell death, and inhibition of NF-κB after partial hepatectomy results in massive hepatocyte apoptosis, worsens liver injury and decreases survival [25]. Enhanced NF-κB activation is also seen in mice that are protected from hepatic I/R following blockade of the receptor for advanced glycation end products [21]. There is evidence suggesting that the impact of APAP toxicity ensues, at least in part, by dramatic modulation of inflammatory and/or regeneration programs [26]. After massive hepatonecrosis induced by APAP overdose, liver regeneration is a vital process for survival after a toxic insult [6,7], and NF-κB activation plays an important role in liver regeneration [8]. Prolonged treatment with NAC impairs liver regeneration, at least partly by inhibiting the NF-κB activation pathway.

Massive hepatocyte necrosis is the predominant feature of APAP-induced ALI. Tissue repair is an important determinant of final outcome of toxicant-induced injury [7], and cyclin D1 is an important cell cycle protein. In the current investigation, our western blot data showed that prolonged NAC treatment markedly decreased the level of cyclin D1 expression in the APAP-challenged liver tissue. The change in cyclin D1 expression was associated with increased serum ALT/AST and delayed liver regeneration in NAC-treated mice receiving APAP, suggesting that prolonged NAC therapy likely inhibits cyclin D1-mediated regeneration pathway, and the reduced cyclin D1 expression might be modulated by decreased NF-κB DNA binding [8].

## Conclusions

Prolonged treatment with NAC impairs liver regeneration in ALI induced by APAP, reevaluation of optimal doses and duration of NAC therapy is needed.

## Key messages

• Prolonged treatment with NAC increases serum ALT/AST in APAP-challenged mice.

• Prolonged treatment with NAC induces hepatocyte vacuolation in APAP-challenged mice.

• Prolonged treatment with NAC delays hepatocyte regeneration in APAP-challenged mice.

• Prolonged treatment with NAC delays liver recovery from APAP hepatotoxicity, reevaluation of optimal doses and duration of NAC therapy is needed.

## Abbreviations

APAP: acetaminophen; ALI: acute liver injury; ALT: alanine aminotransferase; AST: aspartate aminotransferase; EMSA: electrophoretic mobility shift assays; GSH: glutathione; H&E: haematoxylin and eosin; I/R: ischemia-reperfusion; MPO: myeloperoxidase; NAC: N-acetyl-cysteine; NAPQI: N-acetyl-p-benzoquinone imine; NF-κB: nuclear factor κB; PBS: phosphate-buffered saline; SEM: standard error of the mean; TCA: tricarboxylic acid.

## Competing interests

The authors declare that they have no competing interests.

## Authors' contributions

RKY designed the study. All authors participated in the animal handling and procedures. MPF helped to draft the manuscript. All authors read and approved the final manuscript.
